# PD32 Incorporating Environmental Factors Into Health Technology Assessment Submissions: A Scoping Review

**DOI:** 10.1017/S0266462324002952

**Published:** 2025-01-07

**Authors:** Andrew Briggs, Evi Germeni, Jieling Chen

## Abstract

**Introduction:**

A scoping review was conducted to understand which environmental factors are currently being discussed as candidates for inclusion in health technology assessments (HTAs). By mapping the current literature, the results of the scoping review will inform future work to assess the importance policymakers place on aspects of environmental concern.

**Methods:**

The scoping review involved literature searches across three electronic databases (MEDLINE, CINAHL, and PsycINFO), encompassing articles published up to the end of 2023, to identify a starting set of articles. Backward and forward citation searching of relevant studies was then employed to expand the scoping review, with inclusion criteria established to select studies based on the specific focus on HTA. Screening was conducted independently by two reviewers in two stages: title and abstract and then full-text screening. Data extraction involved a structured approach, collating relevant information for thematic analysis.

**Results:**

While the scoping review is ongoing, emerging issues included: methods for incorporating environmental pollution externalities into HTAs; the impact of climate change on health outcomes; intergenerational equity of health outcomes and definitions of sustainability; environmental epidemiology; and the potential for technologies to mitigate the impact of climate change. The ongoing thematic analysis will synthesize the results of the scoping review using mapping and categorization to identify knowledge gaps.

**Conclusions:**

The threat of climate change is the greatest issue faced by HTA in a generation, but this challenge is intersectoral and demands an intersectoral response. This paper identifies environmental concerns that might, in principle, be included within the HTA process. Ultimately, the HTA community must do its part to address the environmental crisis we face and move toward a more sustainable future.

